# The description, measurement with inter‐ and intra‐observer reliability of calcaneal tunnel placement for tendon transfer in Achilles tendon reconstruction

**DOI:** 10.1002/jeo2.70223

**Published:** 2025-06-11

**Authors:** Michael R. Carmont, Tor Kristian Andresen, Fraser Morgan, Katarina Nilsson‐Helander, Elisabeth Ellingsen Husebye

**Affiliations:** ^1^ Department of Orthopaedic Surgery Shrewsbury & Telford Hospital NHS Truat Shropshire UK; ^2^ Department of Orthopaedic Surgery, Sahlgrenska Academy University of Gothenburg Gothenburg Sweden; ^3^ Department of Orthopaedic Surgery Akershus University Hospital Oslo Norway; ^4^ Department of Orthopaedic Surgery Oslo University Hospital Oslo Norway

**Keywords:** calcaneal tunnel, chronic Achilles tendon rupture, tendon transfer

## Abstract

**Purpose:**

A tendon transfer is a common method of treating ankle plantar flexion weakness and tendon end non‐union following chronic Achilles tendon rupture and delayed representation following Achilles tendon re‐rupture. Commonly, the transferred tendon is fixed into a bone tunnel on the postero‐superior surface of the calcaneum close to the distal Achilles tendon insertion. To date, there is no standardised description or measurement of calcaneal tunnel position. The aim of this study is to describe the anatomic location for calcaneal tunnel placement and to determine the reliability of a method of measuring tunnel position and direction within the calcaneum.

**Methods:**

The routine post‐operative lateral ankle radiographs from 40 patients (40 ft) following Achilles tendon reconstruction using tendon transfer into the calcaneum: calcaneal tunnel zone (CTZ), calcaneal tunnel ratio (CTR) and calcaneal tunnel angle (CTA) were tested for reliability using test‐retest between three observers. Additionally, CTR and CTA were compared in cases where a calcaneoplasty was performed or not.

**Results:**

The intraclass correlation coefficient (ICC) of the CTR and CTA was found to be 0.86–0.95 (95% confidence interval [CI]: 0.75‐0.98) and 0.95–0.99 (95% CI: 0.92–0.99), respectively, indicating good and excellent reliability. Patients who received a calcaneoplasty had a significantly greater CTR of 0.74 (0.1) and a lower CTA of 76.1° (10.8) compared to those who did not have a CTR of 0.61 (0.1) and 100.9 (12.4), Diff 95% CI: 0.13 (0.08–0.18) and −25 (−32 to −17), respectively, both *p* < 0.001.

**Conclusions:**

The CTR and CTA were reliable measures for the calcaneal tunnel following Achilles tendon reconstruction using tendon transfer within the limitations of the sagittal radiographic view. When a calcaneoplasty was performed, it resulted in a significantly greater CTR. These measurements should be used to describe calcaneal tunnels rather than a description of tunnel placement to optimise predictive factors following Achilles tendon reconstruction.

**Level of Evidence:**

Level III.

**Abbreviations**
CI: confidence interval
CTA: calcaneal tunnel angle
CTR: calcaneal tunnel ratio
CTZ: calcaneal tunnel zone
FHL: flexor hallucis longus
ICC: intraclass correlation coefficient
N: number
P: probability
PSCT: postero superior calcaneal tubercle
SD: standard deviation

## INTRODUCTION

**Figure 1:**
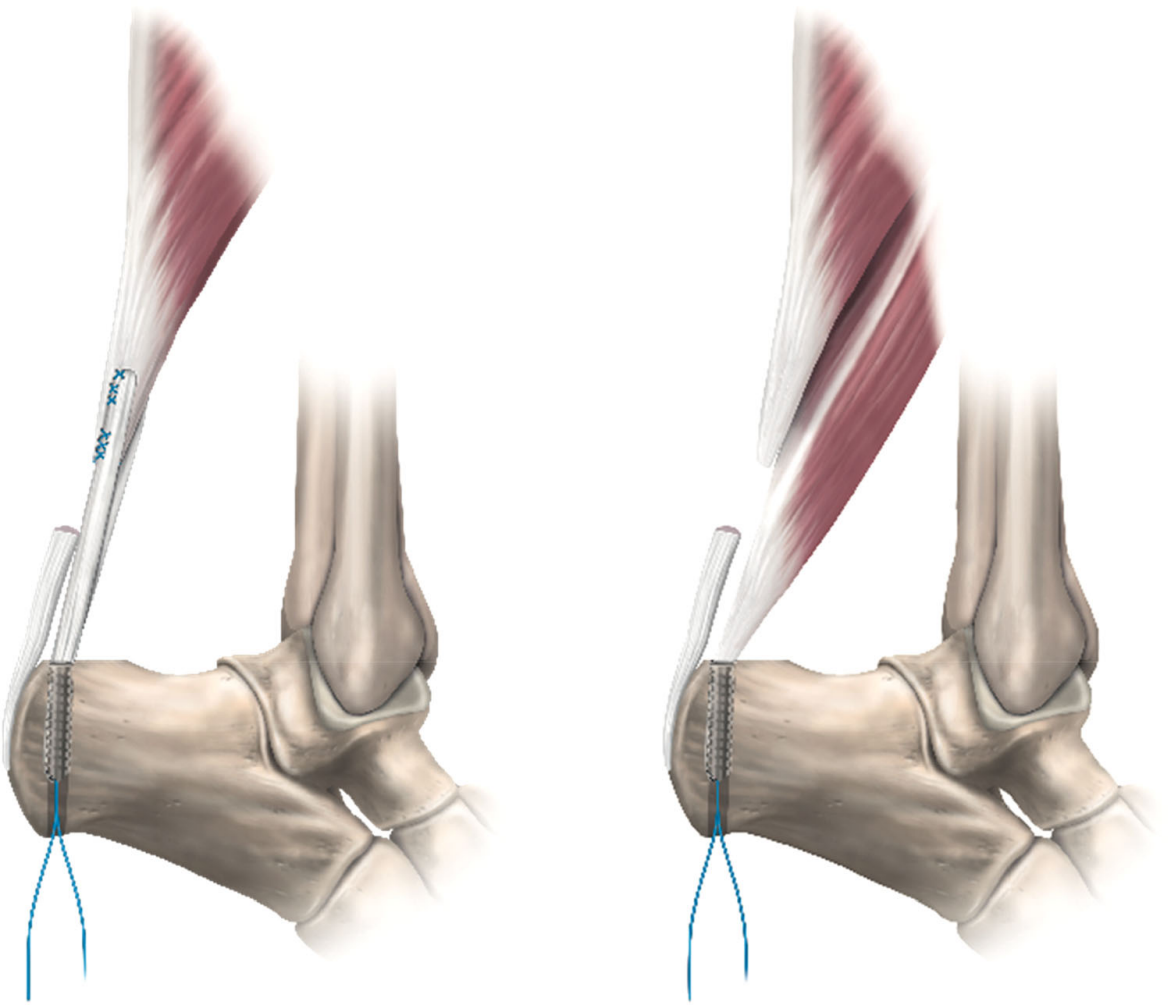
Achilles tendon reconstructions using hamstring (left) and flexor hallucis longus transfer (right).

Achilles tendon reconstruction is a common method of treating ankle plantar flexion weakness and tendon end non‐union following chronic Achilles tendon rupture or delayed representation following Achilles tendon re‐rupture. This procedure aims to optimise patient function by improving ankle plantar flexion strength whilst minimising complications. Reconstruction may be performed by replacement or augmentation of a deficient Achilles tendon through local tendon transfer, such as a local flexor hallucis longus (FHL) and free hamstring tendon transfer. In both these methods, the transferred tendon may be fixed into a bone tunnel on the superior or posterior surface of the calcaneum close to the distal Achilles tendon insertion (Figure 1). The superior surface of the calcaneum is relatively flat and slopes distally from the posterior facet of the subtalar joint before rising to the posterior superior calcaneal tubercle (PSCT) and then descending from the top of the tubercle to the insertion of the Achilles tendon on the posterior surface of the calcaneum [3, 6, 13, 14].

Current reconstruction techniques using FHL and free hamstring tendon transfer place the tunnel close to the insertion of the Achilles tendon, aiming to increase the moment arm of the muscle's contractile force. However, the direct line of pull of the FHL muscle is close to the ankle joint as the tendon passes directly inferiorly beneath the sustentaculum tali [7]. During reconstruction procedures, a galvanoplasty consisting of debridement of the Haglund PSCT may be performed and is thought to allow tunnels to be placed more posteriorly [4] and avoid impingement of the transferred tendon during ankle dorsiflexion. Free hamstring transfer bridges the distal musculotendinous junction, and distally, the graft is placed into the posterior calcaneum close to the Achilles tendon insertion. A double‐strand hamstring tendon produces a thicker graft than a single FHL tendon transfer, and transferred tendons may impinge on the posterior calcaneum. Both techniques show good patient‐reported and functional outcomes [11, 16].

To date, there is no standardised description or measurement of calcaneal tunnel locations. The primary aim of this study was to describe a classification for calcaneal tunnel placement and to determine the reliability of a method of measuring tunnel position and direction on the sagittal radiographic view within the calcaneum. The secondary aim was to see if performing a calcaneoplasty placed the tunnel more posteriorly. The hypothesis was that radiographic measurements would be reliable for determining calcaneal tunnel position, and in future studies, a variable of tunnel position may be used as a predictor of outcome.

## MATERIALS AND METHODS

The routine post‐operative lateral ankle radiographs from patients following Achilles tendon reconstructions for chronic Achilles tendon rupture and delayed representation following Achilles tendon re‐rupture using tendon transfer were included and evaluated. Cases in which there had been a bony avulsion of the Achilles tendon insertion were excluded. Only anonymised radiographic images were compared during this study; therefore, the study was deemed to be a service evaluation and ethical approval was not required. Reconstructions were performed using either an endoscopic FHL tendon [5, 8] or endoscopic assisted free hamstring tendon transfer [4, 15] techniques by three different surgeons (MRC, TKA and EEH), all experienced in Achilles tendon reconstruction.

**Figure 2:**
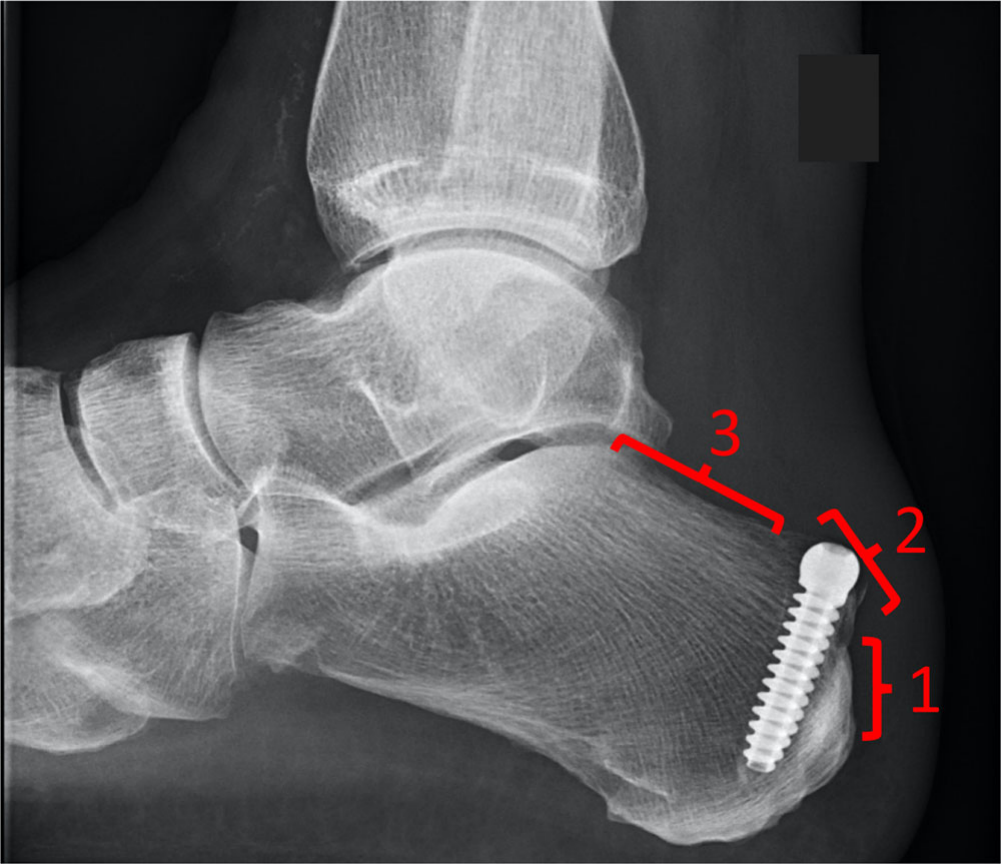
The three descriptive zones for tunnel position. Zone 1 Insertional, Zone 2 Haglund and Zone 3 Proximal.

On the radiographs, the location of the tunnel on the calcaneus was categorised into one of three calcaneal tunnel zones (CTZs) (Figure 2):

- CTZ 1 Insertional zone, at the Achilles insertion, where the posterior cortex is thickened and appears more sclerotic on the radiograph.
- CTZ 2 Haglund zone, at the Haglund, is termed the PSCT until the superior parallel pitch line becomes linear.
- CTZ 3 Proximal zone, along the flat area of the superior parallel pitch line.

**Figure 3:**
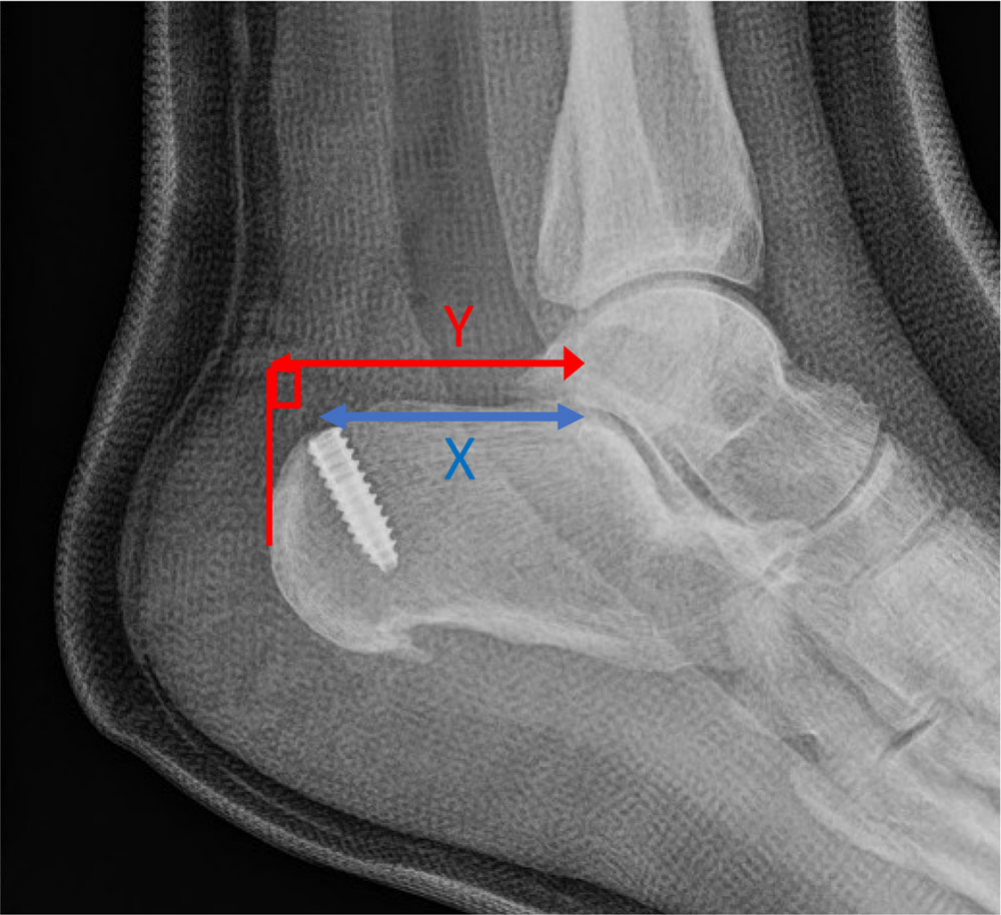
The calcaneal tunnel ratio equals length *X*/length *Y*.

The calcaneal tunnel ratio (CTR) was then determined. The CTR was defined as the distance of the centre of the calcaneal tunnel from the posterior facet of the subtalar joint along the superior parallel pitch line, Length *X*, compared to the length of the calcaneum from the posterior facet of the subtalar joint to the most posterior aspect of the calcaneum, Length *Y* (Figure 3). The CTR is the ratio of length *X* to length *Y* (CTR = *X*/*Y*). If a metal radiopaque screw was used to secure the tendon within the tunnel, the centre of the screw was taken as the centre of the tunnel. If a radiolucent screw was used, then the centre of the radiolucent tunnel was used.

**Figure 4:**
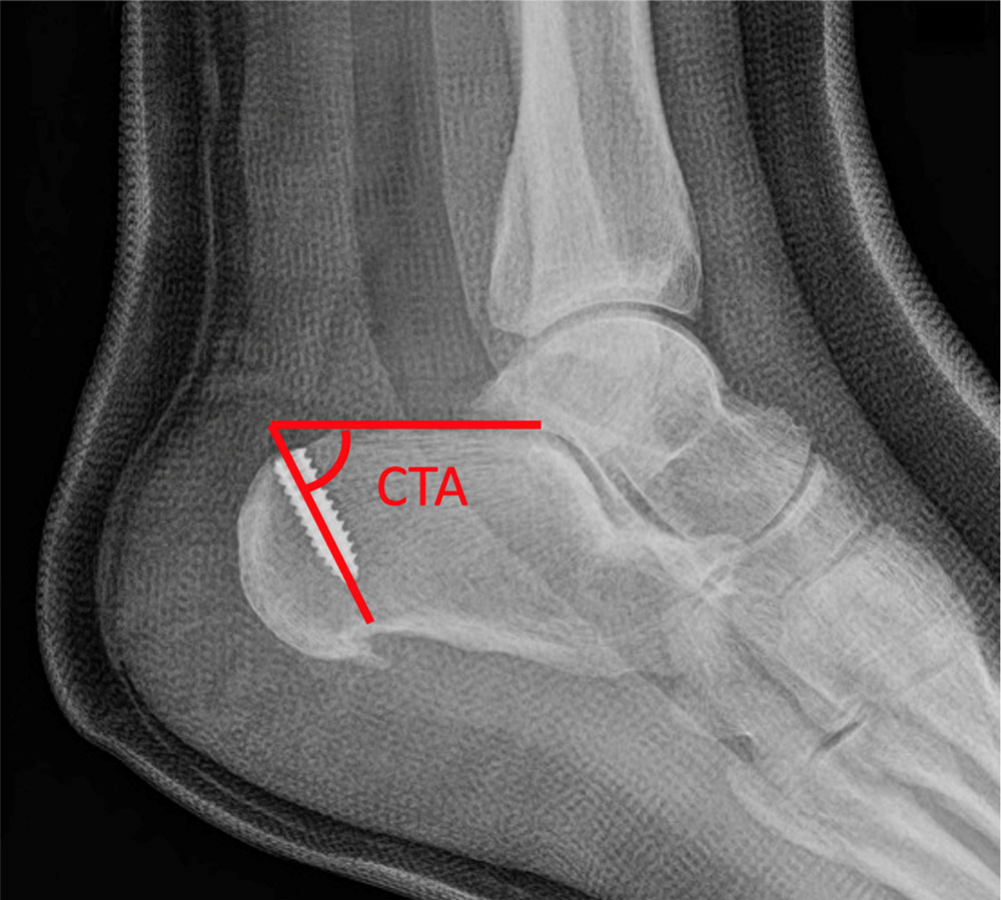
The calcaneal tunnel angle (CTA).

The calcaneal tunnel angle (CTA) was then measured using a 15‐cm arm goniometer with 2° increments. This angle was the acute angle between the superior parallel pitch line and the long axis of the calcaneal screw or tunnel (Figure 4).

After 7 days, the radiographs were categorised once again. The CTZ, CTR and CTA were remeasured to enable the reliability of these measurements to be determined.

The CTR and CTA were compared between radiographs in the following reconstructions, where the surgical technique involved a calcaneoplasty and when no calcaneoplasty had been performed. Additionally, the CTR and CTA were compared when a metal (radio‐opaque) or a bio‐interference (radiolucent) screw was used.

### Statistical analysis

The tunnel zone was described categorically, and the CTR and CTA as continuous values.

Prior to commencing the study, a sample size calculation was performed using a minimum acceptable reliability (intraclass correlation coefficient [ICC]) of 0.60, an expected reliability (ICC): 0.80, a significance level (*α*): 0.05 (two‐tailed) and a Power (1 − *β*) of 80%. Allowing for three raters, an appropriate sample size was determined to be 33 images. According to Koo and Li [10], the ICC estimation, less than 0.5, between 0.5 and 0.75, 0.75 and 0.9 and greater than 0.9 were indicative of poor, moderate, good and excellent reliability, respectively.

The CTR and CTA were considered to have a normal distribution following pictorial representation on a histogram; consequently, comparisons were performed using a Student's *t* test. The tunnel parameters were compared in ankles when a calcaneoplasty or no calcaneoplasty was performed using a Student's *t* test.

## RESULTS

Forty lateral ankle post‐operative radiographs following Achilles tendon reconstruction surgery using tendon transfer of 40 ft were assessed by three raters to determine the inter‐rater reliability.

The descriptive statistics for the CTR and CTA are shown in Table 1.

**Table 1:** Descriptive statistics for the three raters by raters.

	Mean	Standard deviation
Tunnel ratio		
Rater 1 1st	0.66	0.09
2nd	0.65	0.10
Rater 2 1st	0.71	0.10
2nd	0.71	0.10
Rater 3 1st	0.68	0.11
2nd	0.68	0.11
Angle		
Rater 1 1st	90	16.7
2nd	89	16.8
Rater 2 1st	85	16.4
2nd	88	17.3
Rater 3 1st	85	17.9
2nd	85	17.9

Intra‐rater reliability		
	ICC	95% Confidence interval
Tunnel ratio		
Rater 1 1st vs. 2nd	0.95	0.91–0.98
Rater 2 1st vs. 2nd	0.86	0.75–0.92
Rater 3 1st vs. 2nd	0.94	0.88–0.97
Angle		
Rater 1 1st vs. 2nd	0.99	0.98–0.99
Rater 2 1st vs. 2nd	0.95	0.90–0.97
Rater 3 1st vs. 2nd	0.95	0.92–0.98
Zone	Kappa	Absolute agreement
Rater 1 1st vs. 2nd	0.37	93%
Rater 2 1st vs. 2nd	0.73	83%
Rater 3 1st vs. 2nd	0.23	68%

A calcaneoplasty was performed in 21 patients and no calcaneoplasty in 19 patients (Table 2).

**Table 2:** The CTR and CTA of patients who received a calcaneoplasty compared to those who did not receive a calcaneoplasty.

Variable	Calcaneoplasty (*n* = 21)	No Calcaneoplasty (*n* = 19)	Diff (95% CI)	*p*
CTR mean (SD)	0.74 (0.08)	0.61 (0.08)	0.13 (0.08–0.18)	**<0.001**
CTA mean (SD)	76.1 (10.8)	100.9 (12.4)	−25 (−32 to −17)	**<0.001**

Abbreviations: CTA, calcaneal tunnel angle; CTR, calcaneal tunnel ratio; Diff (95% CI), difference in 95% confidence interval; n, number; P, probability; SD, standard deviation.

## DISCUSSION

The most important finding of this study is that the reliability of the CTR and CTA were good and excellent. The CTR was more reliable than the CTZ description of tunnel placement. The CTR uses the same superior parallel pitch line (radiographic greater tuberosity line) to measure calcaneal length as a predictive factor for symptomatic Haglund pathology. Tourné et al. [18] also found excellent intra‐ and‐inter‐observer correlation using this line. These assessment methods of calcaneal tunnel placement can therefore be used as a reliable variable and predictive factor in future comparison studies between different tendon transfer methods for reconstruction following chronic Achilles tendon rupture.

Recent reviews of the management of chronic Achilles tendon rupture have recommended free autologous hamstring transfer for chronic ruptures less than 3 months post‐injury and with a tendon gap more than 5 cm and FHL transfer for chronic ruptures greater than 3 months following rupture [2]. Tendon transfer as reconstruction for Achilles tendon rupture is developing and endoscopic procedures can be technically challenging. Surgeons may choose tunnel position for a variety of reasons including belief in the optimal tunnel position, technical ability, and ease of placement [2, 12]. Open FHL tendon transfer for calcaneal pathology may have placed the calcaneal tunnel in a position of convenience into the calcaneum more anteriorly, and good functional outcomes were reported [19].

The ideal tunnel placement for tendon transfer for Achilles tendon reconstruction is not known. For hamstring reconstruction, the tendon bridges the gap between the proximal tendon end and the calcaneum. Using this technique, it would seem logical to place the tendon in a tunnel within the actual original Achilles insertion. On radiographs, this is noted as the increase in cortical sclerosis on the posterior wall of the calcaneum [13]. Following FHL transfer, the muscle force generated by FHL contraction assists ankle plantar flexion strength and the proximity of the vascular FHL muscle belly, theoretically assisting in the healing of the relatively avascular rupture site facilitating healing [9]. To bring the FHL muscle belly close to the healing site, the transferred tendon should arguably be as close to the Achilles tendon insertion as possible after debriding the deep intervening fascia. This, however, may pose technical challenges when harvesting an adequate free tendon length from within the FHL tenosynovial sheath [20]. Surgeons may compensate by placing a shorter free tendon into a more anterior tunnel, and computer analysis studies have determined this to be a more beneficial location as it is closer to the direct line of pull of the FHL muscle [1].

This study suggests that a calcaneoplasty placed the tunnel at a greater CTR or more posteriorly than when no calcaneoplasty was performed. It must be appreciated that in cases in which a calcaneoplasty was performed by two surgeons, tunnels were drilled and screws placed through either the postero‐medial or a central tendon splitting portal. The other surgeon did not perform a calcaneoplasty, so the tunnel was placed at the Haglund PSCT through a postero‐medial portal. The use of a calcaneoplasty during surgery is controversial and was performed with the aim of placing the calcaneal tunnel as posteriorly as possible during the reconstruction. It would be difficult to place a tunnel and screw at the Achilles insertion using an FHL tendon transfer without performing a calcaneoplasty or a long tendon harvest. Also, if this were performed, the transferred tendon would likely impinge between the Haglund and the distal Achilles tendon. The Haglund protuberance is likely to have a role during gait increasing the lever arm and stretching the Achilles tendon during and just beyond mid‐stance. The calcaneoplasty may prevent this from occurring and reduce dynamic strength during walking. The primary aim of this study was to determine the reliability of the CTR and CTA measurements and further studies looking at the relationship between tunnel position and reconstruction using both techniques are required before firm conclusions can be made.

The strengths of this study are that the zones are well‐defined and consist of the Achilles insertion, the region of the Haglund, and the calcaneum proximal to the Haglund. The study follows a standard methodology to determine reliability ratings. One of the factors to consider for the CTR measurements is that the ratio will not be affected by projection as with any change in obliquity. The length of the calcaneum will appear to change with projection; however, the ratio will remain the same. For research purposes, lateral ankle weight‐bearing radiographs could be used to standardise projections, minimising this influence on the CTA. This would, however, require sufficient rehabilitation to have elapsed to enable patients to be fully weight‐bearing on a plantigrade ankle. Conversely, the good reliability confirms that the ratio method is suitable for intra‐operative fluoroscopy to determine tunnel location.

The CTZ and CTR have been based on the lateral radiograph and may be best suited to tunnels placed predominantly in the sagittal plane, although they may be placed in the transverse or coronal plane [17]. The line of pull for a transverse tunnel is likely to be within the same zone or slightly more proximally than those described in this study. This study did not consider angulation within the coronal plane, but it took place on the calcaneal axial view. In the author's experience, the calcaneal axial view is much harder to obtain. It would be considerably more challenging to standardise position and alignment due to inversion/eversion of the hindfoot intra‐operatively or in a cast with the ankle in plantar flexion and sub‐talar joint in inversion. Computer tomography image reconstruction would be ideal, but there are considerations regarding increased radiation exposure and finance.

One limitation of the zonal descriptive method is that the Haglund or PSCT may or may not have been excised during reconstruction. In this study, the Haglund zone was considered to start when the bone cortex rises from the flattened surface of the calcaneum posterior to the posterior facet of the subtalar joint. The Achilles insertion on the lateral radiograph may similarly be difficult to identify but was located at the thickened sclerotic cortical bone on the posterior calcaneum [13].

A further consideration is that a hand‐held goniometer was used in this study rather than the features of a digital imaging system due to the challenges of importing images into patient archiving systems between different hospitals. The use of a computerised technique may make the measurements even more reliable.

## CONCLUSION

The measurement of the CTR and CTA had good and excellent inter‐observer reliability within the limitations of a sagittal radiographic view. When a galvanoplasty was performed, this resulted in a significantly greater CTR. The determination of the CTR and CTA is recommended in future studies of tendon transfer following Achilles tendon reconstruction.

## AUTHOR CONTRIBUTIONS

Michael R. Carmont had the idea for the study. Michael R. Carmont, Tor Kristian Andresen, Elisabeth Ellingsen Husebye and Katarina Nilsson‐Helander performed the observer testing. Fraser Morgan performed the randomisation and blinding independently. All co‐authors were involved in the writing of the paper and all co‐authors have seen and approved the final version of the paper.

## CONFLICT OF INTEREST STATEMENT

M. R. Carmont is a member of the Editorial Board of *Knee Surgery Sports Traumatology and Arthroscopy* journal. The remaining authors declare no conflicts of interest.

## ETHICS STATEMENT

The study was deemed to be service evaluation, and ethical approval was not required.

## Data Availability

The data involved in this study will be available upon reasonable request to the Authors.
